# Localization and activity of lipoxygenase in the ovule of *Larix kaempferi* (Lamb.) Carr. during female gametophyte maturation

**DOI:** 10.1007/s00497-024-00507-9

**Published:** 2024-07-26

**Authors:** Aleksandra Seta-Koselska, Ewa Szczuka, Mateusz Koselski

**Affiliations:** 1 Department of Plant Physiology and Biotechnology, Institute of Biological Sciences, The John Paul II Catholic University of Lublin, 20-708 Lublin, Poland; 2 Department of Cell Biology, Institute of Biological Sciences, Maria Curie-Skłodowska University, 20-033 Lublin, Poland; 3 Department of Plant Physiology and Biophysics, Institute of Biological Sciences, Maria Curie-Skłodowska University, 20-033 Lublin, Poland

**Keywords:** Lipoxygenase, *Larix kaempferi*, Female gametophyte, Immunolocalization, LOX activity

## Abstract

**Key message:**

Lipoxygenase activity and localization vary throughout the development of Larix kaempferi ovules, with the highest enzyme activity observed in ovules at the cellular stage and the most intense immunogold reaction noted at the mature archegonium stage of gametophyte development.

**Abstract:**

Lipoxygenases are a family of oxidoreductases with a significant role in biological systems, widespread in living organisms e.g. mammals, fish, corals, plants, mosses, algae, fungi, yeasts, and bacteria. Lipoxygenase activity in plants leads to the formation of phytooxylipins, i.e. signaling molecules, which play a crucial role in many significant physiological processes such as male and female gametophyte maturation, germination and seedling growth, pathogen resistance, abiotic stress response, fruit ripening, and senescence. The activity and localization of lipoxygenase change during plant growth and development. The localization of lipoxygenase in a developing ovule of *Larix kaempferi* was analyzed using the immunogold labeling method, and the activity was determined spectrophotometrically with linolenic acid as a substrate. Among the investigated stages, the immunogold reaction was the most intense at the mature archegonium stage in the ovule. Lipoxygenase was found in all parts of the *L. kaempferi* ovule. The largest number of immunogold particles was detected in the integument cells of all the analyzed stages of ovule development. Only one isoform of lipoxygenase with an optimum at pH 8 was active in the ovules during female gametophyte maturation. The highest enzyme activity was determined at the cellular stage, whereas the mature archegonium stage was characterized by its lowest level, which means that LOX activity in developing ovules of the Japanese larch is not correlated with the number of antibody-labeled molecules of the enzyme.

## Introduction

Larch trees (*Larix*) (family Pinaceae) are economically important and inherent components of the forest boreal flora. These extremely durable trees with resistance to environmental factors enrich the species composition of high-latitude and -altitude forest areas of Eurasia and North America. Moreover, due to their intensive growth, relatively modest soil requirements, high resistance to low temperatures and wind, and relatively high resistance to air pollution, including industrial emissions, *Larix* species are valuable in cultivation for commercial purposes (Chylarecki 1991). The undeniable ornamental advantages of *Larix* species determine their use for decoration of parks and gardens.

Among more than the 10 species of the *Larix* genus inhabiting the northern hemisphere, the Japanese larch (*Larix kaempferi*) stands out for its tough and durable wood. This species forms a stable population in its natural habitat, i.e. volcanic mountains in Japan. As a monoecious gymnosperm plant, *L. kaempferi* produces male (pollen) and female (seed) cones growing among radially arranged clusters of deciduous grey- or blue-green needle-like leaves. The characteristics of seed-cones, especially at the initial phases of development, and some aspects of flowering, including megasporogenesis, were described earlier (Seta-Koselska et al. 2015).

The favorable tree growth rate, disease resistance, and finally the reproductive and evolutionary success are a result of many factors, including the proper functioning of enzymes involved in the key development processes. One of the enzyme families with divergent functions in the plant is constituted by commonly occurring lipoxygenases (LOXs; EC 1.13.11.12). These enzymes have been studied in some prokaryotic organisms, for example cyanobacteria (Beneytout et al. 1989), and in many representatives of all the kingdoms of eukaryotes, including animals (Schewe et al. 1986). The long list of organisms begins with lower eukaryotes such as non-filamentous baker’s yeast *Saccharomyces cerevisiae* (Schechter and Grossman 1983), more structurally advanced fungi (Hamberg 1986), algae (Zimmerman and Vick 1973), and mosses (Stumpe et al. 2006). Soybean lipoxygenase is the most often studied enzyme among the LOXs in angiosperms (Baysal and Demirdöven 2007).

Lipoxygenases are an omnipresent family of non-heme, non-sulfur oxidoreductases, containing iron or manganese as a cofactor and catalyzing the hydroperoxidation of polyunsaturated fatty acids (PUFAs). Substrates of lipoxygenases are PUFAs containing cis, cis-1,4-pentadiene moieties; in the plant kingdom, these are linoleic acids (LA, 18:2ω6), linolenic acid (ALA, 18:3ω3), and roughanic acid (16:3ω3) in plants in which the biosynthesis of glycolipids occurs mainly in plastids (Göbel and Feussner 2009). According to regiospecificity, plant lipoxygenases are classified as 9- and 13-LOXs, or LOXs with dual specificities: 9/13-LOX or 13/9-LOX—depending on the product formed principally (Hughes et al. 2001). Products of hydroperoxidation are metabolized to various oxylipins by several enzymes organized into 9-LOX and 13-LOX pathways, including allene oxide synthase (AOS), hydroperoxide lyase (HPL), divinyl ether synthase (DES), peroxygenase, epoxy alcohol synthase, alkyl hydroperoxide reductase, and LOX itself (Howe and Schilmiller 2002). The 9-LOX pathway plays an essential role in plant defense against microbial pathogens, whereas the 13-LOX pathway leads to production of such signaling compounds as jasmonates and green leaf volatiles (GLVs). Most of the lipoxygenases reported in plants belong to 13-LOXs (Viswanath et al. 2020).

Lipoxygenases have been found in various plant tissues and subcellular parts where they are engaged in diverse functions. LOXs act as vegetative storage proteins (VSPs) mainly in seeds but also in non-seed tissues; during germination, different sets of LOXs are involved in mobilization of lipids (Porta and Rocha-Sosa 2002). Upon herbivore attack, LOXs initiate antiherbivore oxidative shift, which induces both direct and indirect oxidative injury to the herbivore as well as production of signaling molecules engaged in plant defense (Kaur et al. 2014). The 9-LOX pathway product, 9-HOT (9-hydroxy-10,12,15-octadecatrienoic acid), has been found to limit pathogen invasion through modification of cell walls in *Arabidopsis thaliana* infected with *Pseudomonas syringae* (Vellosillo et al. 2013). 9-LOX products also play a key role in hypersensitive response (HR)—a process limiting pathogen spread via induction of rapid plant cell death around the infection site (Davoine et al. 2006). Lipoxygenase is involved in regulation of response to abiotic stress caused by drought, high salinity, heavy metals, low or high temperature, light, and wounding (Padilla et al. 2014; Hou et al. 2015; Liu et al. 2016; Kotapati et al. 2017; Prasad et al. 2017; Mazur et al. 2018). During senescence, the level of LOX activity increases, which leads to formation of superoxide radicals, lipid peroxidation, and membrane damage (Ahmad and Tahir 2018; Del Ángel-Coronel et al. 2018). In senescing leaves, 13-LOX present in chloroplasts binds to the plastid envelope and oxidizes membrane fatty acids, which leads to the formation of holes in the plastids and leakage of stromal constituents to the cytosol (Springer et al. 2016). Numerous data show that, during senescence, specific LOX genes were highly expressed, which was accompanied by an increase in the level of superoxide radicals (Liu and Han 2010; Ling et al. 2018; Mazur et al. 2018; Wu et al. 2018). 13-LOX also participates in the production of jasmonates (JAs), i.e. a class of phytohormones widely distributed in higher plants. Jasmonic acid and its derivates are signal molecules, which control many physiological processes such as root growth, growth of reproductive organs, seed germination, plant senescence, plant responses to abiotic and biotic stress, and the biosynthesis of various metabolites (Ghorbel et al. 2021; Wan and Xin 2022). With the participation of other hormones, such as abscisic acid, auxin, cytokinin, ethylene, gibberellic acid, and salicylic acid, jasmonates balance growth and defense processes, thereby facilitating plant acclimation to changing environmental conditions (Liu and Timko 2021) Jasmonates regulate the reproductive development of plants. Jasmonic acid plays a pivotal role in pollen maturation and dehiscence as well as female reproductive development (Stintzi and Browse 2000; Li et al. 2001; Park et al. 2002; He et al. 2021). According to numerous studies, the JA signaling pathway is involved in fertility regulation in angiosperms (Li et al. 2022; Wan and Xin 2022; Huang et al. 2023). Lipoxygenases, which are crucial at early steps in the biosynthesis of jasmonates, influence the formation and development of sexual structures in angiosperms; however, little is known about its role in the development of generative organs in gymnosperms.

Although such enzymes as LOX are a frequent object of scientists’ interest, there are no data on the subcellular localization of LOX and its connection with the ovule development at the maturation phases of the female gametophyte. Therefore, we decided to undertake the research to address this issue. Moreover, it was an additional challenge to study gymnosperms with a much more complex process of female gametophyte formation than angiosperms.

## Materials and methods

### Plant material

The seed-cones of *Larix kaempferi* at various stages of ovule development were collected twice a week from April to June from the Botanical Garden of UMCS in Lublin (Poland).

### Fixation of plant material for light and electron microscopy

Seed scales with ovules were isolated and fixed in 3% formaldehyde (freshly prepared from paraformaldehyde) with 2% glutaraldehyde dissolved in 0.1 M phosphate buffer (pH 7.3) for 24 h at 4°C. The plant material was rinsed several times in PBS and 0.5 M NH_4_Cl in PBS, dehydrated in ethanol and acetone series, and saturated with mixtures of acetone and LR White resin (Sigma, USA) and finally with pure LR White resin. The seed scales were transferred to capsules, embedded in LR White resin, and polymerized at 60°C overnight. The LR White resin-embedded plant material was cut into semi-thin sections (1.5 μm) for light microscopy and thin sections (70–75 nm) for electron microscopy using a Reichert Ultracut S ultramicrotome. The semi-thin sections were collected on a microscope slide and stained with toluidine blue.

### Immunolocalization of LOX

A modified method described by Szczuka et al. (2006) was used for immunogold-labeling. Thin sections were collected on formvar coated nickel grids, treated with aqueous 0.56 M sodium periodate for 30 min, washed in deionized water, treated with 10 M HCl for 10 min, and then washed with water for 5 min. The samples were incubated in 1% BSA in 0.1 M PBS (pH 7.3) for 45 min at room temperature and then with PBS-BSA containing rabbit anti-LOX polyclonal serum (Agrisera, Sweden) diluted 1/800 for 1 h. After triplicate 10-min washing with PBS-BSA, the sections were incubated with goat anti-rabbit immunoglobulins conjugated with 10 nm gold particles (GAR-gold, Sigma, USA) diluted 1/50 in PBS-BSA for 40 min at room temperature. The samples were washed in PBS and then in deionized water. The control samples were incubated with GAR-gold, omitting anti-LOX antiserum. In order to visualize intracellular structures in the biological material, the sections were stained with 2% uranyl acetate and Reynold’s reagent. The samples were examined and photographed with a Zeiss LEO 912 AB (Carl Zeiss, Germany) electron microscope. The intensity of the immunogold reaction was determined by counting gold particles on the surface of cross-sections of entire cells or fragments of cells. The measurement of the analyzed surface was carried out using a standard function of the program (AnalySiS 3.0) supporting a microscope camera.

### Determination of LOX activity

The activity of LOX was measured spectrophotometrically at 234 nm as described earlier (Skórzyńska-Polit and Krupa 2003). The reaction mixture contained 0.2 M boric acid buffer (pH 6–10) or 0.2 M citratic buffer (pH 4–5), 25 μl of plant extract, and linoleic acid as a substrate in 3 ml of the final volume. The reaction was carried out at 30°C for 4 min. The LOX activity was expressed as an absorbance increase per mg of protein per minute. The protein concentration was measured according to Bradford (1976) using BSA as a standard.

## Results

### Localization of lipoxygenase in the ovule of *Larix kaempferi* during female gametophyte maturation

#### Localization of LOX in the ovule with the gametophyte at the cellular stage

**Fig. 1:**
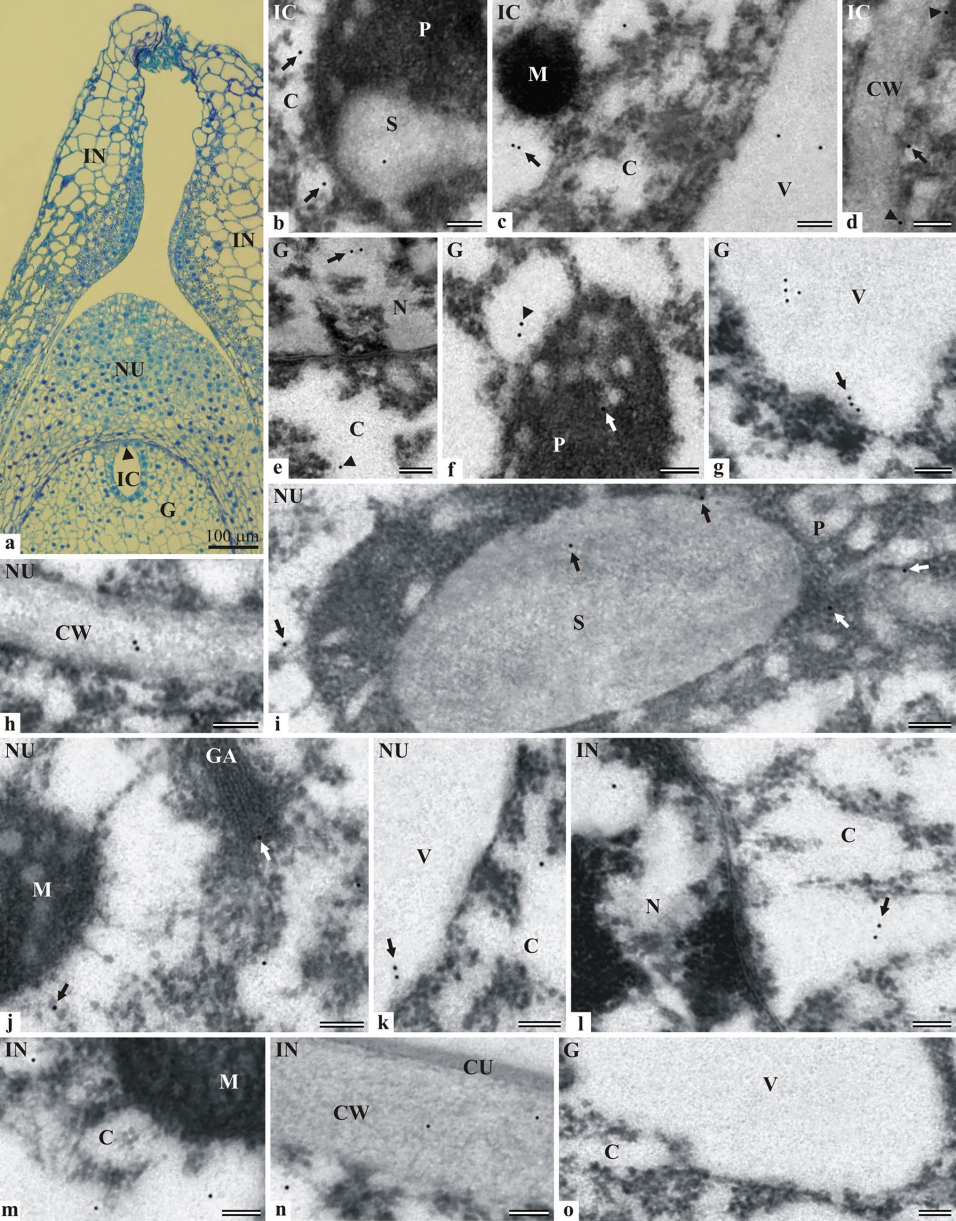
Immunolocalization of lipoxygenase in the ovule of *L. kaempferi* at the cellular development stage of the gametophyte: fragments of the initial cell (**b**–**d**), gametophyte (**e**–**g**), nucellus (**h**–**k**), and integument (**l**–**n**). Longitudinal section showing the ovule at the cellular stage of the gametophyte. Semi-thin section stained with toluidine blue. Archegonium initial cell with the nucleus located in the micropylar part (arrowhead) (**a**). Fragment of the plastid with a starch grain. Single immunogold particles in the cytoplasm near the plastid (arrows) and in the starch grain (**b**). Portion of the cytoplasm with a mitochondrion and a fragment of the vacuole with single or paired (arrow) immunogold particles (**c**). Fragment of the cell wall separating the initial cell from the other gametophyte cells. Single immunogold particles in the cytoplasm of IC near the cell wall (arrow) and at the plasmalemma (arrowheads) (**d**). Portion of the nucleus with paired immunogold particles in the nucleoplasm (arrow); single immunogold particle in the cytoplasm (arrowhead) (**e**). Fragment of a plastid with a single immunogold particle (arrow) and surrounding cytoplasm with paired immunogold particles (arrowhead) (**f**). Portion of the cytoplasm and fragment of the vacuole of the gametophyte cell. More numerous immunogold particles grouped inside a vacuole and near the tonoplast (arrow) (**g**). Fragment of the cell wall between nucellus cells with paired immunogold particles (**h**). Fragment of a plastid with a starch grain and surrounding cytoplasm with single immunogold particles (arrows) (**i**). Immunogold particles near the mitochondrion and Golgi apparatus (arrows) in the cytoplasm of the nucellus cell (**j**). Fragment of the cytoplasm with a single immunogold particle and a vacuole with paired (arrow) immunogold particles (**k**). Fragment of the integument cell with a single immunogold particle in the nucleoplasm and paired immunogold particles in the cytoplasm (arrow) (**l**). A Portion of the cytoplasm with a mitochondrion fragment of the integument cell. Single immunogold particles near the organelle are visible (**m**). Fragment of the integument cell wall bordering the external environment with a thick cuticle layer. Single gold particles inside the cell wall and in the cytoplasm near the cell wall (**n**). Control reaction. Fragment of the vacuole surrounded by the cytoplasm of the gametophyte cell (**o**). TEM bar 100 nm. Abbreviations: cell wall–CW, cuticle–CU, cytoplasm–C, gametophyte–G, Golgi apparatus–GA, initial cell–IC, integument–IN, mitochondrion–M, nucellus–NU, nucleus–N, plastid–P, starch grain–S, vacuole–V

At the cellular stage (Fig. 1a), the gametophyte consisted of highly-vacuolated cells with diverse sizes and shapes. The nucleus in the archegonium initial cell was located at the micropylar pole of the ovule, and the center of this cell was occupied by a large vacuole. The developing initial cell was surrounded by a single layer of small tapetum cells, which had regular shapes on the section, a large nucleus, and dense cytoplasm. In the nucellus at this stage, dividing cells were very rarely observed, and most of the cells exhibited a varied degree of vacuolation. The integument cells showed significant diversity, e.g., the cells adjacent to the micropylar channel had a small size, regular shape, and dense cytoplasm, whereas the cells located behind them and forming the external part of the integument were larger and contained numerous plastids. The smaller cells building the internal part of the integument often contained greenish stained resin or tannins. In the upper part, the integument was strongly elongated and closed the micropylar channel.

The localization of lipoxygenase was studied in all parts of the Japanese larch ovule. Small amounts of immunogold particles indicating the presence of lipoxygenase were found in the cytoplasm of the micropylar side of the initial cell of the archegonium. They were located near the amyloplast and inside the starch grain contained therein (Fig. 1b). Single immunogold particles were also present near the mitochondrion (Fig. 1c), in the wall of the initial cell of the archegonium, and near the plasmalemma (Fig. 1d). Paired particles were found in the vacuole (Fig. 1c). In the cells of the gametophyte, immunogold particles indicating the presence of the enzyme were detected in the nucleus (Fig. 1e), inside the plastid, and in the cytoplasm near this organelle (Fig. 1f). The immunogold particles in the vacuole were more numerous and often formed groups (Fig. 1g). In the cells of the nucellus, the immunogold particles indicating the presence of lipoxygenase were observed in the cell wall (Fig. 1h), the amyloplast, the cytoplasm near the plastid (Fig. 1i), the mitochondrion, Golgi folded membranes (Fig. 1j), and the vacuole (Fig. 1k). In the integument cells, enzyme molecules were revealed in the nucleoplasm (Fig. 1l), the cytoplasm near the mitochondrion (Fig. 1m), and in the vicinity or within the cell walls (Fig. 1m). No immunogold particles were found in the cuticle layer covering the integument. After the control reaction, no immunogold particles indicating the presence of LOX were visible in the gametophyte cell fragment (Fig. 1o).

#### Localization of LOX in the ovule with the gametophyte at the young archegonium stage

**Fig. 2:**
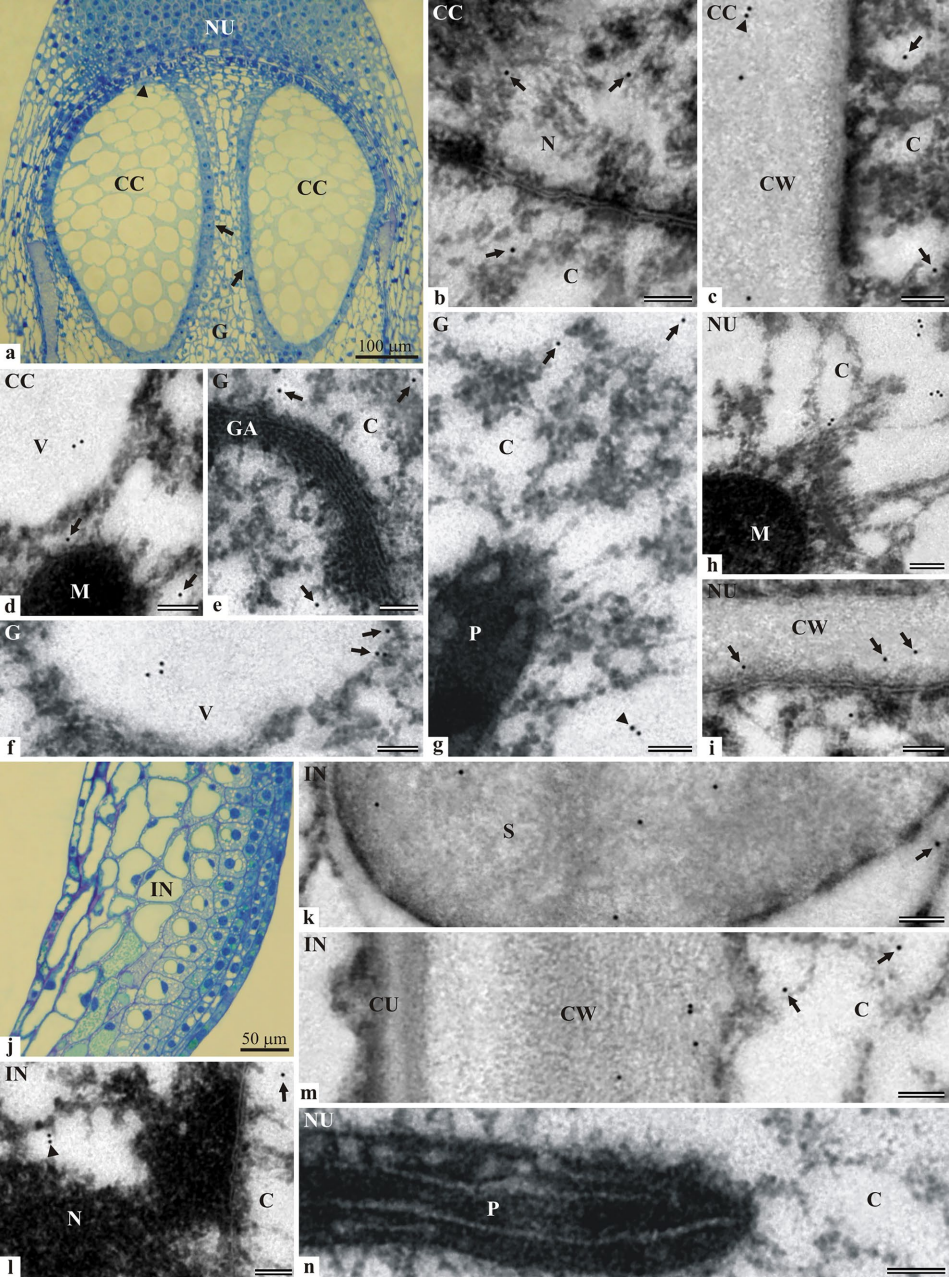
Immunolocalization of lipoxygenase in the ovule of *L. kaempferi* containing archegonia at the early stage of development: **a** and **j**—LM (staining with toluidine blue), **b**–**i** and **k**–**n**—TEM. Longitudinal section taken through the micropylar part of the ovule containing the female gametophyte nucellus with the central cells of two archegonia at the young development stage. The nucleus located in the micropylar part of the central cell (arrowhead). Each archegonium surrounded by a single layer of tapetum cells (arrows) (**a**). Fragment of the nucleus and cytoplasm of the central cell. Visible nuclear envelope (membrane) and poorly condensed chromatin. Single immunogold particles in the area of the nucleoplasm and in the cytoplasm near the nucleus (arrows) (**b**). Fragment of the cell wall surrounding the central cell. Single immunogold particles (arrows) visible in the cytoplasm and single or paired immunogold particles (arrowhead) in the area of the cell wall (**c**). Portion of the central cell with visible fragments of the mitochondrion and the vacuole. Single immunogold particles (arrows) are localized in the cytoplasm near the mitochondrion. Two gold particles visible in the vacuole next to each other (**d**). Portion of the cytoplasm of the gametophyte cell with the Golgi apparatus. Single immunogold particles in the cytoplasm in the vicinity of the Golgi apparatus (arrows) (**e**). Part of the gametophyte cell with a fragment of the vacuole. Group of three immunogold particles visible inside the vacuole and at the tonoplast (arrows) (**f**). Portion of the cytoplasm with a plastid fragment. Single (arrows) or paired (arrowhead) immunogold particles in the cytoplasm (**g**). Portion of the cytoplasm with a fragment of the mitochondrion with clustered immunogold particles (**h**). Fragment of the cell wall between the nucellus cells. Single immunogold particles visible in the cell wall area (arrows) (**i**). Fragment of the ovule integument. Note the differences in the anatomical structure of the external (on the right) and internal (on the left) parts of integument (**j**). Fragment of the starch grain with single dispersed immunogold particles. Single immunogold particle in the cytoplasm near the starch grain (arrow) (**k**). Portion of the nucleus and the cytoplasm of the integument cell. Single immunogold particle in the cytoplasm near the nucleus (arrow) and paired immunogold particles in the nucleus with condensed chromatin (arrowhead) (**l**). Fragment of a thick wall covered by a cuticle layer separating the integument cell from the external environment. Within the cell wall, single or paired immunogold particles near the edge bordering the cytoplasm; single particles (arrows) in the cytoplasm (**m**). Control reaction. Portion of the nucellus cell cytoplasm with a visible fragment of the plastid (**n**). TEM bar 100 nm. Abbreviations: cell wall–CW, central cell of archegonium–CC, cuticle–CU cytoplasm–C, gametophyte–G, Golgi apparatus–GA, integument–IN, mitochondrion–M, nucellus–NU, nucleus–N, starch grain–S, plastid–P, vacuole–V

The archegonium initial cell divided asymmetrically into a large central cell and a small primary neck cell. After this mitotic division, the central cell continued to enlarge, which was accompanied by very strong vacuolization of the cytoplasm. The longitudinal section through the Japanese larch ovule containing a central cell in each of the two archegonia is shown in Fig. 2a.

After the intensive growth phase, the central cell reached about 450 μm in length and 230 μm in width. The small nucleus of the central cell was located in the micropylar part immediately under the plasmalemma. In the cross-section, the four-sided tapetum cells surrounding each central cell were small and filled with dense cytoplasm and contained relatively large nuclei. In contrast, the other cells of the gametophyte were characterized by an irregular shape, different sizes, and small nuclei. No dividing cells were found in the nucellus, but the number of highly vacuolated and degenerated cells increased. In the integument, a clear difference in the structure between the layers is visible (Fig. 2j). The cells lining the micropylar canal were smaller and had a more regular shape in the longitudinal section, dense cytoplasm, and a relatively large nucleus. Toward the outer side of the integument, there were layers formed by larger cells with a nearly centrally located nucleus and one large central vacuole. In the outer part of the integument, there were enormous (compared to others) irregularly shaped cells with a large central vacuole and a peripherally located cell nucleus. Numerous cells containing resin were present in the integument. The localization of lipoxygenase in the Japanese larch ovule at the young archegonium stage was studied in the central cell (Fig. 2b–d) and other cells of the gametophyte (Fig. 2e–g) as well as the cells of the nucellus (Fig. 2h and j) and the integument (Fig. 2k–m). In the central cell, single immunogold particles were observed in the nucleus and in the perinuclear cytoplasm (Fig. 2b), near and inside the wall (Fig. 2c), in the vacuole and cytoplasm near the mitochondrion (Fig. 2d). In the gametophyte cells forming the tapetum layer, single immunogold particles were observed in the cytoplasm near such organelles as the Golgi apparatus (Fig. 2e) and the plastid (Fig. 2g), while a group of three immunogold particles was often observed in the vacuoles beside the single particles near the tonoplast (Fig. 2f). In the cells of the nucellus, mostly clusters of three immunogold particles were found in the cytoplasm (Fig. 2h) and single immunogold particles were detected in the cell wall (Fig. 2i). In the integument cells, the immunogold particles were found in the cytoplasm around the organelles and in starch grains (Fig. 2k). Single or paired immunogold particles were found in the nucleus and perinuclear cytoplasm (Fig. 2l). The presence of the enzyme was revealed both in the area of the internal wall between the integument cells and in external walls separating the integument from the environment, but no immunogold particles were noticed in the cuticle layer (Fig. 2m). No immunogold particles were visible after the control reaction (Fig. 2n).

#### Localization of LOX in the ovule with the gametophyte at the mature archegonium stage

**Fig. 3:**
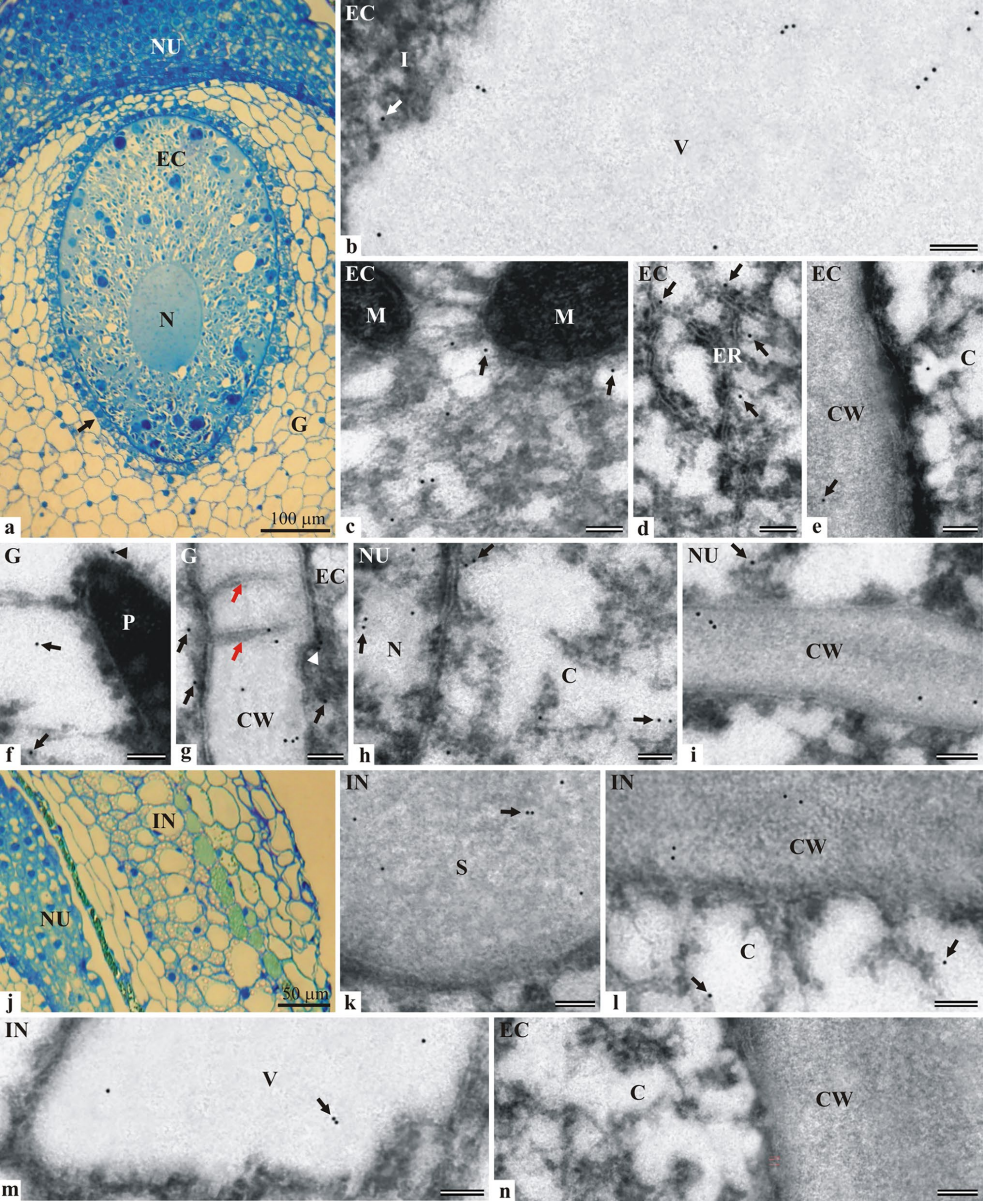
Immunolocalization of lipoxygenase in the ovule parts of *L. kaempferi* at the mature archegonium developmental stage: in the egg cell (**b**–**e**), the cells of gametophyte (**f**,**g**), nucellus (**h**,**i**), and integument (**k**–**m**); **a** and **j**—LM (staining with toluidine blue), **b**–**i** and **k**–**n**—TEM. Longitudinal section showing the micropylar part of the ovule with an egg cell surrounded by a single layer of tapetum cells (arrow) (**a**). Part of an autophagic vacuole of the egg cell containing single or grouped immunogold particles; single gold particle in the vacuolar inclusion (arrow) (**b**). Part of the organelle-rich perinuclear cytoplasm with visible fragments of mitochondria. Immunogold particles in the cytoplasm near the organelles (arrows) (**c**). Portion of perinuclear cytoplasm with a visible endoplasmic reticulum. Immunogold particles near the membranes of reticulum cisterns (arrows) (**d**). Fragment of the egg cell wall. Single particles in the cytoplasm and inside the cell wall (arrow) (**e**). Portion of the cytoplasm with a fragment of the plastid. Single immunogold particles in the cytoplasm (arrows) and at the outer plastid membrane (arrowhead) (**f**). Fragment of the cell wall between the egg and the gametophyte cell with visible plasmodesmata (red arrows). Single (arrows) or paired (arrowhead) immunogold particles at the plasmalemma of the egg and the gametophyte cells; single or grouped immunogold particles within the cell wall (also inside the plasmodesmata) (**g**). Part of the cytoplasm and the nucleus of the nucellus cell with single or paired (arrows) immunogold particles (**h**). Fragment of the cell wall between the cells of the nucellus. Cluster of three immunogold particles in the cell wall; single particles in the wall and in the cytoplasm (arrow) (**i**). Longitudinal section of a fragment of the *L. kaempferi* ovule integument (**j**). Part of the integument cell with single or paired (arrow) immunogold particles in the area of the starch grain (**k**). Fragment of the cell wall and the cytoplasm. Single gold particles in the cytoplasm (arrows) and paired gold particles in the cell wall (**l**). Part of the vacuole with visible single or paired (arrow) immunogold particles (**m**). Control reaction. Fragment of the egg cell containing the cytoplasm and cell wall (**n**). TEM bar 100 nm. Abbreviations: cell wall–CW, cytoplasm–C, egg cell–EC, endoplasmic reticulum–ER, gametophyte–G, inclusion–I, integument–IN, mitochondrion–M, nucellus–NU, nucleus–N, starch grain–S, plastid–P, vacuole–V

After the period of intensive growth of the central cell, the phase of intensive synthesis of cytoplasmic components and the accumulation of spare substances began. The vacuoles in the central cell disappear and storage materials in the form of small and large inclusions appear in the cytoplasm. During the third ten days of May, the central cell divided asymmetrically into a small, lenticular ventral canal cell and an enormous egg cell. The stage of a mature archegonium with a visible egg cell is shown in Fig. 3a. The oval egg cell reached a very large size (about 500 μm in length and 300 μm in width). During development, the nucleus of the egg cell also enlarged (150 µm/100 µm) and moved from the micropylar pole towards the center of the cell. Mitochondria and other cellular organelles gathered around the nucleus. Numerous small and large inclusions were evenly dispersed in the cytoplasm of the egg cell. Some inclusions were hydrolyzed in autophagic vacuoles. At this stage, the egg cell was ready to fertilization. The egg cell was surrounded by a single layer of tapetum cells showing a higher degree of degradation than at an earlier stage of development. The other cells of the gametophyte were differentiated in their size and shape and all contained a very small nucleus located near the walls. At the examined stage, some nucellus cells shrunk, filled with dense cytoplasm, and tightly adhered to each other. The ovule integument was built of a highly differentiated cell layer. The inner part of the integument consisted of layers of smaller, narrow cells, in which green-stained resin was noticeable. Behind them, towards the outer surface, the cells were larger, elongated, and highly vacuolated. The internal part formed parenchymal cells, among which the central layer was a strip of resin-containing cells (Fig. 3j).

At the mature archegonium stage, the location of lipoxygenase was studied in the egg cell (Fig. 3b–e) and in the cells of the gametophyte (Fig. 3f and g), as well as the nucellus (Fig. 3h and i) and the integument (Fig. 3k–m). In the egg cell, the immunogold particles were detected in cytoplasmic inclusions. In the autophagic vacuoles, groups of three molecules of the enzyme revealed by immunogold particles were often observed (Fig. 3b). Numerous immunogold particles were found in the organelle-rich perinuclear region of the cytoplasm. Lipoxygenase was found in the vicinity of mitochondria (Fig. 3c) and near endoplasmic reticulum cisterns (Fig. 3d). Single immunogold particles were observed in the cytoplasm near the cell wall of the egg cell and inside the wall (Fig. 3e). In the gametophyte cells, the presence of lipoxygenase was detected in the vacuoles, cytoplasm near plastids (Fig. 3f), and mitochondria (not shown in any picture). Single or grouped immunogold particles were present in the cell wall between the egg and the tapetum cell with numerous plasmodesmata and in the cytoplasm near the cell wall. Single gold particles were also found inside plasmodesmata (Fig. 3g). The nucellus cells contained numerous immunogold particles indicating the presence of lipoxygenase in the nucleus and in the cytoplasm near the nuclear envelope (Fig. 3h). Immunogold particles were also observed in the cytoplasm near the mitochondria, the endoplasmic reticulum cisterns and in the cell wall. In the wall between the nucellus cells, immunogold particles occurred individually or in pairs (Fig. 3i). In the integument cells, the gold particles were located in the starch grain (Fig. 3k), the cell wall (Fig. 3l), and the vacuole (Fig. 3m). In the latter structure, two gold particles were often observed next to each other. Lipoxygenase was also located in the cytoplasm near the cell wall and organelles. The control reaction did not show any immunogold particle in the fragment of the egg cell section (Fig. 3n).

### Intensity of immunogold reaction and activity of LOX in the ovule of *Larix kaempferi* during female gametophyte maturation

**Fig. 4:**
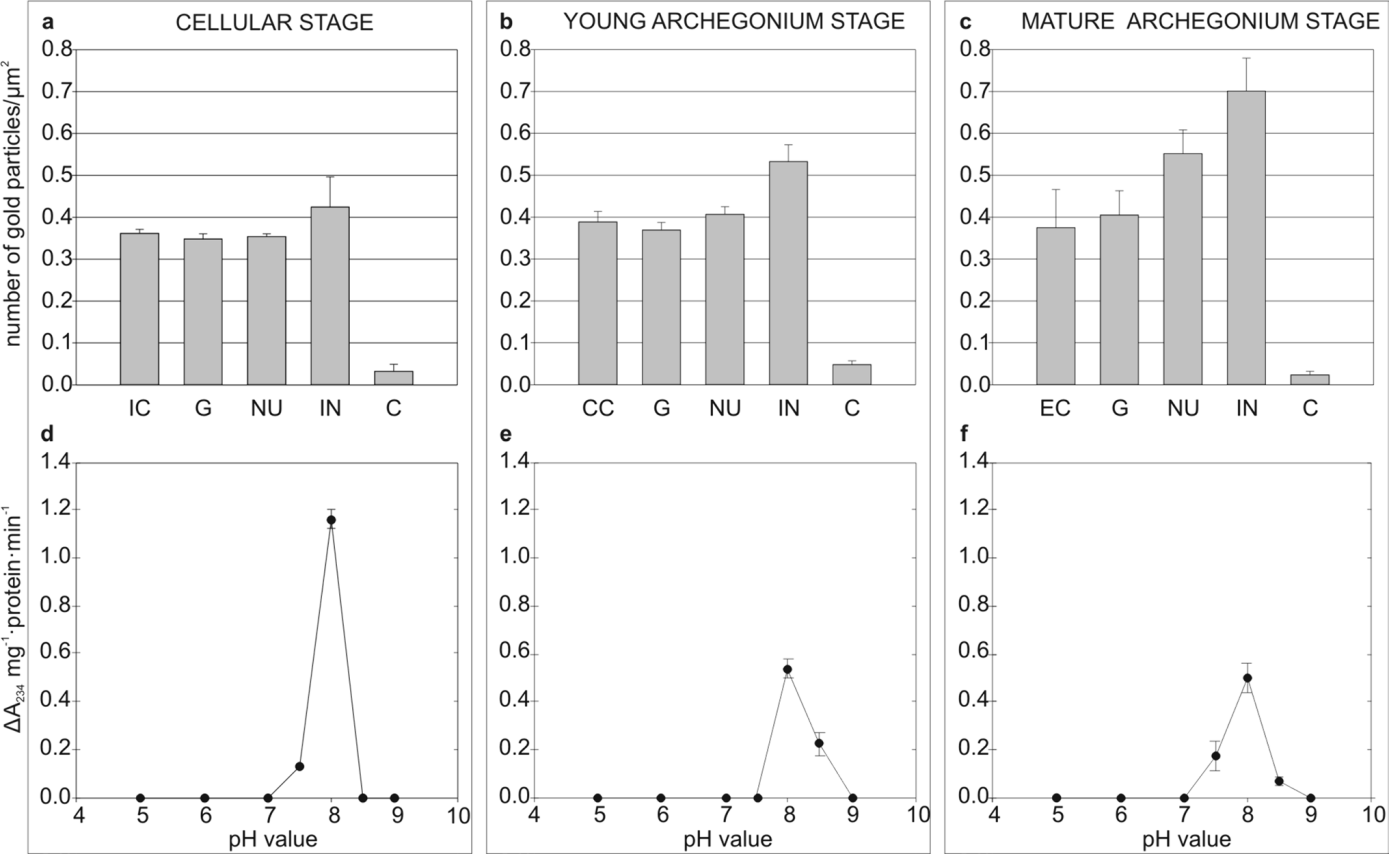
Intensity of immunogold reaction and LOX activity in the *L. kaempferi* ovule during female gametophyte maturation. Amount of immunogold particles in the ovule at the cellular stage (**a**). Amount of immunogold particles in the ovule at the young archegonium stage (**b**). Amount of immunogold particles in the ovule at the mature archegonium stage (**c**). LOX activity in the ovule at the cellular stage (**d**). LOX activity in the ovule at the young archegonium stage (**e**). LOX activity in ovule at the mature archegonium stage (**f**). Abbreviations: archegonium initial cell–IC, central cell–CC, egg cell–EC, gametophyte–G, nucellus–NU, integument–IN, control–C

At the cellular stage, similar low intensity of the immunogold reaction was observed in the examined parts of the ovule with the gametophyte. The largest number of immunogold particles was found in the integument cells (Fig. 4a). Similarly, the immunogold reaction was not intense in the ovule of *L. kaempferi* at the young archegonium stage (Fig. 4b). The highest number of gold particles indicating the presence of lipoxygenase was found in the integument, and the lowest number was exhibited by the gametophyte. There were slight (insignificant) differences in the amount of gold particles between the central cell and the other cells of the gametophyte and the nucellus. At the mature archegonium stage, the immunogold reaction was the most intense among the investigated stages (Fig. 4c). Most of the immunogold particles were found in the integument cells, whereas their lowest abundance was exhibited by the egg cell. At this stage, greater variation in the amount of immunogold particles between the individual elements of the ovule was noted.

At all the investigated stages, only one lipoxygenase isoform with an optimum at pH 8 was active. At the cellular stage, the enzyme activity was the highest among all the investigated stages and was equal to 1.16 ΔA_234_ mg^−1^·protein·min^−1^ (Fig. 4d). A significant decrease in the activity of lipoxygenase to 0.53 ΔA_234_ mg^−1^·protein·min^−1^ was observed in the ovules of the Japanese larch at the young archegonium stage (Fig. 4e). The activity of the enzyme at the mature archegonium stage did not change significantly, compared with the previous stage, and was equal to 0.495 ΔA_234_ mg^−1^·protein·min^−1^ (Fig. 4f).

## Discussion

The Japanese larch (*Larix kaempferi*), whose ovules were used as a material in this study, is one of eleven species representing the genus *Larix* and one of only about 1100 species of gymnosperms currently occurring around the globe (Christenhusz and Byng 2016). Although gymnosperms have a very modest number of species constituting only 1% of plant diversity, they play a unique role in the world’s flora. The number of gymnosperm species that are currently regarded as threatened with extinction, estimated at almost 40% as high risk, is a concern for scientists (Forest et al. 2018). Beside other gymnosperms, larches are the dominant plants in the natural boreal forests of the Northern Hemisphere (Crepet and Niklas 2009). Among the larch species, the Japanese larch is becoming more popular, especially in the countries of northern Europe. Other larch species, e.g. *Larix decidua*, *L. kaempferi*, and *L. occidentalis*, have also been investigated due to their poor seed quality and production (Hall and Brown 1977; Kosiński 1987; Owens et al. 1994; Slobodnik 2000; Slobodník and Guttenberger 2005). The authors analyzed the symptoms and causes of these unfavorable phenomena, focusing mainly on reproductive processes that lead to the production of seeds (Philipson 1996; Slobodnik and Guttenberger 2000; Slobodník and Guttenberger 2005).

The localization of lipoxygenase was studied at the subcellular level in the *L. kaempferi* ovule at three phases of female gametophyte development, i.e. the cellular gametophyte with the initial cell of the archegonium, the young archegonium, and the mature egg cell-containing archegonium. In addition to the cellular gametophyte, such parts of the ovule as the nucellus surrounding the gametophyte and the integument were investigated. The enzyme was detected using the precise immunogold method in the cells of all these parts of the developing ovule. LOX was revealed in the cytoplasm near such organelles as plastids, mitochondria, and the nucleus. Moreover, it was found inside the nucleus, the vacuole, and in the area of the starch grain. Additionally, the presence of the enzyme was observed in the area of the cell walls and near the plasmalemma. The presence of LOX in these structural elements of cells is justified by its participation in a wide range of processes taking place in plants (Viswanath et al. 2020). The cells of developing plant organs responsible for reproduction are very active, especially during the formation of the gametophyte. Intensive processes of synthesis and disintegration take place in these tissues (Pereira and Coimbra 2019).

Most often, the immunogold particles revealing the presence of LOX appeared singly and rarely in clusters of two or three. The single immunogold particles indicating the localization of LOX were observed inside undifferentiated plastids or in the close vicinity to the plastid envelope membranes in the cells of all structural ovule elements. The presence of LOX in these areas may be justified by the roles played by this enzyme. Considering the presence of lipoxygenase in ovule cells, it is necessary to take into account the structure of these plant organs responsible for reproduction. Plastids are present in the cells of the developing ovule at the juvenile stage of development. This is evidenced by their simple structure without plastoglobules or grana, which are characteristic elements of chloroplasts. It is known that the envelope membranes of differentiated plastids, such as chloroplasts, are involved in the regulation of plant cell metabolism and membrane biogenesis (Blée and Joyard 1996). Other authors underline the participation of enzymes contained in the envelope membranes of chloroplasts isolated from spinach (*Spinacia oleracea* L.) leaves in the catalysis of the rapid breakdown of fatty acid hydroperoxides. A multifunctional lipoxygenase with fatty acid hydroperoxide cleaving activity has been found in the moss *Physcomitrella patens* (Senger et al. 2005). In a more recent report, it has been proved that LOX-13 is involved in the destruction of chloroplasts during leaf senescence (Springer et al. 2016). During this process, the mechanism of lipoxygenase action consists in breaking down unsaturated fatty acids localized in the plastid envelope membrane. The perforations produced in this way allow the chloroplast content to flow out. This mechanism works in both natural and artificially MeJA (methyl jasmonate) induced senescence. Cell degeneration processes take place during the development of the ovule, e.g. during the elimination of non-functional megaspores or the breakdown of nucellus cells during the intensive growth and development of the female gametophyte. However, the function of LOX localized near the plastids in the ovule cells of the female gametophyte at the maturation stage cannot be fully explained by the typical aging mechanism. It is likely that LOX molecules in or around plastids are inactive. It can be assumed that they will be used at the later stages, leading to the formation of the seed.

The LOX particles were also localized near the tonoplast, the plasma membrane, and the nuclear envelope. The participation of LOX in membrane degradation by induction of the collapse of these cell membranes was suggested in a study of tapetal cells in *Gagea lutea* (Szczuka et al. 2006). In the anther, tapetal cells undergo programmed cell death (PCD) in order to use the materials during microsporogenesis and pollen grain formation. Such a process may be connected with the acquisition of nucellus materials necessary for the development and maturation of the female gametophyte in the larch ovule. In addition, intensive development of the gametophyte is accompanied by an increase in the number of cells. In certain phases of mitotic and meiotic divisions, the nuclear envelope breaks down (Pradillo et al. 2019), and it can be assumed that LOX plays an essential role in this process.

In addition to the localization of lipoxgenase at the subcellular level with the immunogold method, the intensity of the immunogold reaction was determined by counting the immunogold particles indicative of the presence of lipoxygenase. Inherently, the intensity of this reaction in all the studied parts of the ovule was low and very similar in the individual structures of the *L. kaempferi* developing ovule. Slightly higher numbers of the particles were observed in the integument cells at the three investigated phases of ovule development, but their largest number was present at the mature archegonium stage. At all stages, the lowest immunogold reaction intensity was observed in the gametophyte cells and, especially, in the egg cell. Unexpectedly, the intensity of the immunogold reaction did not coincide with the lipoxygenase activity in the examined parts of the ovule. The highest activity of the enzyme was found in the ovule with the cellular gametophyte. The LOX activity significantly decreased in the subsequent phases of ovule development. However, low and similar LOX activity was demonstrated in the ovules with both young and mature archegonia. Such a lack of correlation between the intensity of the immunogold reaction and LOX activity was noted at the earlier stages of ovule development in *L. kaempferi* (Seta-Koselska et al. 2015). Similarly to the authors’ suggestion, in the ovule with the maturing gametophyte, some LOX molecules can be inactive, and this form of the enzyme performs the function of storage protein. This assumption has been proved with respect to vegetative lipoxygenases (VLXs) functioning as a temporary nitrogen storage in soybean seedlings (Fischer et al. 1999). Moreover, the authors showed that the storage LOXs and VLXs, called C and D, exhibit enzymatic activity in soybean seeds. No doubt, the inactive form of LOX can fulfill such a function in the ovules, which further develop into seeds.

Our results showed the presence of only one active isoform of LOX at all the investigated stages. Additionally, the enzyme was active with an optimum at pH 8. Two peaks (one with the optimum at pH 8 and the other at pH 7) of LOX activity occurred in the youngest ovule of Japanese larch (Seta-Koselska et al. 2015). These results indicate the appearance of two active isoforms. These two isoforms of LOX appeared in the ovule with a triad of megaspores in the nucellus. One of the triads of megaspores, in *Larix* usually the chalazal megaspore, becomes the initial cell of the megagametophyte. In comparison to this crucial stage of future gametophyte development, the maturation stages investigated by us are connected with the development of the gametophyte and preparation of the ovule to pollination and egg cell fertilization. Nevertheless, both these phases of ovule development are essential for processes that determine the development of normal seeds.

Noteworthy, the vast majority of the data in the available literature present LOXs occurring in angiosperms. The results presented by us in this study may constitute an incentive for further research on lipoxygenases in gymnosperms.

## Conclusions

- The development of the ovule and female gametophyte of *Larix kaempferi* is typical for representatives of the Pinaceae family.
- Studies of lipoxygenase activity and the localization of the enzyme using the immunogold labeling method indicate the functioning of the lipoxygenase pathway in all the cells of the analyzed structures of the developing ovules of *Larix kaempferi*.
- The presence of lipoxygenase was demonstrated in the cytoplasm, vacuoles, plastids, starch grains, the cell wall and, to a lesser extent, in the cell nucleus.
- In the cytoplasm, lipoxygenase molecules were present singly or in groups near the mitochondria, endoplasmic reticulum cisterns, dictyosomes and close to the plasmalemma and tonoplast.
- In all the examined developmental stages of Japanese larch ovules, one isoform of lipoxygenase with an optimum at pH 8 was active.
- The activity of lipoxygenase is not correlated with the amount of enzyme-locating immunogold particles found in cells of Japanese larch ovules, which indicates that some LOX molecules can be inactive and perform a function of storage protein.

### Author contribution statement

Conceptualization ASK, ES; methodology ES, ASK; investigation ASK, MK; data analysis ASK, MK; writing—original draft preparation ASK, ES; writing—review and editing ES, MK; visualization ASK, MK. All authors have read and agreed to the published version of the manuscript.

## Data Availability

The data that support the findings of this study are available from the corresponding author upon reasonable request.
